# A comparative study of three models to analyze the impact of air pollutants on the number of pulmonary tuberculosis cases in Urumqi, Xinjiang

**DOI:** 10.1371/journal.pone.0277314

**Published:** 2023-01-17

**Authors:** Yingdan Wang, Chunjie Gao, Tiantian Zhao, Haiyan Jiao, Ying Liao, Zengyun Hu, Lei Wang

**Affiliations:** 1 College of Public Health, Xinjiang Medical University, Urumqi, Xinjiang, China; 2 Department of Infection Prevention and Control, Puyang People’s Hospital, Puyang, Henan, China; 3 State Key Laboratory of Desert and Oasis Ecology, Xinjiang Institute of Ecology and Geography, Chinese Academy of Sciences, Urumqi, Xinjiang, China; 4 Department of Medical Engineering and Technology, Xinjiang Medical University, Urumqi, Xinjiang, China; Università degli Studi Gabriele d’Annunzio Chieti Pescara: Universita degli Studi Gabriele d’Annunzio Chieti Pescara, ITALY

## Abstract

In this paper, we separately constructed ARIMA, ARIMAX, and RNN models to determine whether there exists an impact of the air pollutants (such as PM_2.5_, PM_10_, CO, O_3_, NO_2_, and SO_2_) on the number of pulmonary tuberculosis cases from January 2014 to December 2018 in Urumqi, Xinjiang. In addition, by using a new comprehensive evaluation index DISO to compare the performance of three models, it was demonstrated that ARIMAX (1,1,2) × (0,1,1)_12_ + PM_2.5_ (lag = 12) model was the optimal one, which was applied to predict the number of pulmonary tuberculosis cases in Urumqi from January 2019 to December 2019. The predicting results were in good agreement with the actual pulmonary tuberculosis cases and shown that pulmonary tuberculosis cases obviously declined, which indicated that the policies of environmental protection and universal health checkups in Urumqi have been very effective in recent years.

## 1 Introduction

Tuberculosis is a chronic respiratory disease mainly caused by *Mycobacterium tuberculosis* (*M*. *tuberculosis*), which can invade many organs of the human body, the most common is pulmonary tuberculosis (PTB) infection [1]. A total of tuberculosis with 833 thousand cases from China has been reported in 2020 [2]. The incidence of PTB in Xinjiang is the highest in China, with an incidence of 210.75/per100,000 from 2014 to 2018, and shown an overall increasing trend [3]. Urumqi, the capital of the Xinjiang, has the higher incidence of PTB (60/100,000) [4] with the incidence of new smear-positive PTB (14.31/100,000) than the national average level [5].

PTB is transmitted by breathing in droplet nuclei with single *Mycobacterium tuberculosis* in air from the cough or sneeze of active PTB infected persons [6]. It has been shown that there is a strong association between air pollutants and PTB incidence [7], for instance, PM_2.5_, with the features of being small, light, toxic, and suspended in the air for a long time, which may facilitate the transmission and development of PTB [8]. Oxidative stress and immune inflammatory response produced by human body could increase the risk of PTB [9] when PM_2.5_ is deposited in the lungs. Because O_3_ is the pollutant produced by NO_2_ ultra-violet light, it can worsen lung function, exacerbate airway inflammatory responses and affect lung ventilation. Zhu *et al*. [10] studied the correlation between PTB incidence and PM_10_ and NO_2_ in Chengdu from 2010 to 2015 by using a distributed lag non-linear model. A generalized additive model was by Huang *et al*. [11] applied to analyze the effect of PM_2.5_, PM_10_, and O_3_ on PTB incidence in Wuhan from 2015 to 2016. The positive correlation between the air quality index in Beijing and the incidence of PTB was analyzed in [12].

Urumqi, the economic, cultural, scientific, and transportation center of Xinjiang, is an important central city in Northwest China. It’s surrounded on three sides by mountains and under the control of cold Mongolian high pressure in winter. This special valley topography would make the airflow difficult to flow horizontally and air pollutants difficult to diffuse and dilute. What’s more, the heating period in Urumqi is lasting up to 180 days, and the main energy is coal, which can produce a large number of air pollutants. It has a certain impact on the incidence of PTB. Several studies have shown the effect of air pollution on the PTB cases in Urumqi. For example, an ARMA (1, (1, 3)) + model was established to analyze the correlation between air pollutants and the incidence of PTB in Urumqi from 2014 to 2017 and found that the higher concentration of O_3_, the higher PTB incidence [13]. Yang *et al*. [14] used a generalized additive model to analyze the relationship between air pollutants and PTB incidence and it was indicated that the combined effect of PM_10_ and NO_2_ had the greatest impact on the incidence of tuberculosis.

In order to estimate the relationship between variables described disease dynamics, one of the classical statistical approaches is the use of auto-regressive integrated moving average (ARIMA) model. This model is easy to be constructed, only requires intrinsic variables, and has relatively high prediction accuracy [15]. Therefore, it has been widely applied in the prediction of PTB incidence. As a generalized and improved ARIMA model, ARIMAX (Auto-regressive Integrated Moving Average-X) model can take into consideration the dependence on time series and the disturbance of random fluctuations. By incorporating the exogenous variables into the ARIMA model, ARIMAX can effectively improve the prediction accuracy and accurately predict the short-term trend of the disease, and had been often applied in the prediction of some diseases, such as influenza [16], hand-foot-mouth disease [17], new crown pneumonia [18] and mumps [19], etc. Tuo *et al*. [20] separately established ARIMA and ARIMAX models to analyze and predict the monthly influenza cases in Urumqi from 2013 to 2016. An ARIMAX model was by Li *et al*. [21] applied to analyze the impact of meteorological factors on the incidence of PTB in Kashgar from 2005 to 2014.

Except for time series analysis, deep learning models, such as Recurrent Neural Network (RNN) model, Long-short Term Memory (LSTM) model, Bi-Directional LSTM model and Gate Recurrent Unit (GRU) model, etc. can also be widely applied to forecast disease incidence [22, 23]. RNN is the most common deep learning model which is proposed by Saratha Sathasivam in 1982 [22]. The internal structure of RNN model is simpler, and it can select fewer parameters than other deep learning models with complex structures (LSTM, GRU) [24]. LSTM and GRU models, variants of RNN model, could effectively capture the semantic association between long-term sequences, and alleviate the phenomenon of gradient disappearance [25–27]. Moreover, GRU could also reduce the network parameters compared with LSTM, and converge with faster speed [27]. Particularly, RNN is a sub-class of artificial neural network using hidden variables as a memory to capture temporal dependencies between system and control variables, which is more suitable for handling time series data [28]. So it is widely used to predict the incidence of various diseases, such as hepatitis [29], hands-foot-and-mouth disease [30], COVID-19 [31, 32], dengue fever [33]. For example, Xia *et al*. [29] showed that RNN model is significant to forecast the Hepatitis incidence and have the potential to assist the decision-makers in making efficient decisions for the early detection of the disease incidents. Wang *et al*. [30] predicted the number of hands-foot-and-mouth cases of enterovirus A71 subtype in Beijing from 2011 to 2018 by using RNN model. Kumar *et al*. [31] constructed RNN model to forecast the counts of newly infected COVID-19 individuals, losses, and cures. In [32], RNN model was confirms to have a better predicting performance compared with LSTM and GRU models. Vicente *et al*. [33] applied RNN model to determine whether there was a correlation between the confirmed cases of dengue fever and climate variables.

Therefore, based on the above discussion, the impact of air pollutants (O_3_, PM_2.5_, PM_10_, SO_2_, CO, and NO_2_) on the number of PTB cases in Urumqi was investigated by using the ARIMA model, a multivariate time series ARIMAX and RNN model. And, the best lag orders of the impact of each air pollutant on the PTB cases were determined by the cross-correlation test and spearman rank correlation test. In addition, a new comprehensive assessment index DISO [34, 35], which can circumvent the contradiction of performance result (such as better consistency but worse bias for the same model), was applied to select the optimal one in these three models. Finally, this optimal one was used to predict the number of PTB cases in Urumqi from January to December 2019, which provides a theoretical basis for the prevention and control of PTB in Urumqi.

## 2 Material and methods

### 2.1 Data collection

The monthly PTB cases in Urumqi from January 2014 to December 2018 were obtained from Public Health Scientific Data Sharing Center (https://www.phsciencedata.cn/Share/ky_sjml.jsp?id=f90892b6-c000-48fe-a73e-a4c6db172385. Assessed 4 Dec 2021) and Health Commission of Xinjiang Uygur Autonomous Region (http://wjw.xinjiang.gov.cn/hfpc/jbjcypj/nav_list.shtml. Assessed 4 Dec 2021).

The monthly average values of air pollutants from January 2014 to December 2018 were obtained from the China National Environmental Monitoring Centre (https://www.aqistudy.cn/historydata/monthdata.php?city=%E4%B9%8C%E9%B2%81%E6%9C%A8%E9%BD%90. Assessed 6 Dec 2021), including average PM_2.5_ (*μ*g/m^3^), average PM_10_ (*μ*g/m^3^), average SO_2_ (*μ*g/m^3^), average CO (*μ*g/m^3^, average NO_2_ (*μ*g/m^3^) and average O_3_ (*μ*g/m^3^).

### 2.2 Time series analysis

#### 2.2.1 Model specification

In this paper, it is assumed that both the response series {*y*_*t*_} and the series of input variables {*x*_1*t*_}, {*x*_2*t*_}, …, {*x*_*kt*_} are stationary, the regression model for the response series and the series of input variables is constructed as follows:

$$y_{t}=\beta_{0}+\overset{k}{\underset{i=1}{\sum }}\frac{\Theta_{i}\left(B\right)}{\Phi_{i}\left(B\right)}B^{l_{i}}x_{it}+\varepsilon_{t},$$

where *β*_0_ is the constant of this model, Θ_*i*_(*B*) is the *p*_*i*_-order auto-regressive polynomial of *x*_*it*_ (*i* = 1, …, *k*), Φ_*i*_(*B*) is the *q*_*i*_-order moving average polynomial of *x*_*it*_ (*i* = 1, …, *k*), *B* is the delay operator, *l*_*i*_ is the delay order of {*x*_*it*_}, {*ε*_*t*_} is the series of regression residual and is stationary because both the response series and the input variables series are stationary. By using ARMA model to extract the relevant information in {*ε*_*t*_}, the following regression model can be established:

$$y_{t}=\beta_{0}+\overset{k}{\underset{i=1}{\sum }}\frac{\Theta_{i}\left(B\right)}{\Phi_{i}\left(B\right)}B^{li}x_{it}+\frac{\Theta \left(B\right)}{\Phi \left(B\right)}a_{t},$$

where *β*_0_, (*B*), Φ_*i*_(*B*), *B* and *l*_*i*_ have the same meaning as the above equation. Θ(*B*) is the moving average polynomial of {*ε*_*t*_}, Φ(*B*) is the auto-regressive polynomial of {*ε*_*t*_}, *a*_*t*_ is a white noise series with the mean 0.

#### 2.2.2. Model discernment, parameters estimation, and model diagnosis

The stationarity of the response series (PTB cases series) and the input variable series (air pollutants series) were tested by the Augmented Dickey-Fuller (ADF) test. If they were non-stationary, the nonseasonal difference and seasonal difference methods were applied to stabilize the series. In addition, we identified parameters (p, q, P, and Q) to establish plausible models by referring to the auto-correlation function (ACF) and partial autocorrelation function (PACF) plots based on the stationary series. Firstly, we determined the seasonal part parameters (P and Q) and then nonseasonal part parameters (p and q) for the ARIMA model. Secondly, for the selected models, the least squares method was applied to estimate the parameters and the Ljung-Box test was applied to examine the residuals. Only when the residuals of the selected models were white noise, indicating that the model completely extracted information from the original data. Finally, the optimal ARIMA model was determined according to the lowest corrected Aiken’s information criterion (AIC) and Bayesian information criterion (BIC) [23].

#### 2.2.3. Inclusion of air pollutants

The corresponding residual white noise sequence of each air pollutant variables was obtained by the optimal ARIMA model selected in subsection 2.2.2. And the optimal ARIMA model of each air pollutant variables were used as filter to obtain the residual white noise sequence of the PTB cases, so the pre-whitening process was completed [36]. Moreover, the best lag orders of the impact of each air pollutant on the PTB cases were determined by the cross-correlation function (CCF) of residual white noise. And those air pollutants variables (*P* < 0.05) which were significantly correlated with the PTB cases were included in the multivariate ARIMA model, it was mean that the ARIMAX models were constructed.

### 2.3. Recurrent neural network model

**Fig 1:**
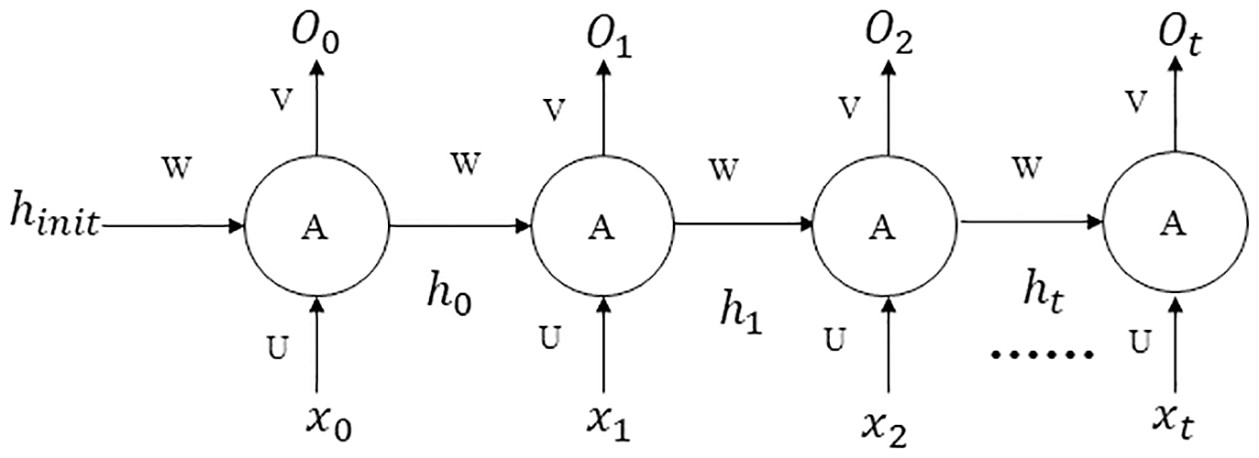
The unfolding diagram of the forward propagation of the RNN.

RNN could be used to describe the relationship between the current output of a sequence and the previous information, which usually consists of an input layer, a hidden layer, and an output layer. RNN is different from the traditional artificial neural network in that it adds connections between the neurons in the hidden layer based on layers fully connected. The unfolding diagram of the forward propagation of the RNN was shown in Fig 1, and the corresponding model is as follows:

$$h_{t}=f\left(Ux_{t}+Wh_{t-1}\right),$$

$$O_{t}=f\left(Vh_{t}\right),$$

where *x*_*t*_ represents input at time *t*, *h*_*t*_ represents the corresponding hidden state at time *t*, *U* and *W* are the weight of *x*_*t*_ and *h*_*t*_, respectively, *O*_*t*_ represents output at time *t*, where *V* represents the weight of *O*_*t*_, *f* is any activation function. Therefore, the input of the RNN hidden layer includes not only the output of the input layer, but also the output of the upper time hidden layer. The data was divided into training set, testing set, and predicting set in a 6:2:2 ratio. In each RNN model, the learning rate was set to 0.05, 0.1, and 0.2 and the dimensions of the hidden layer to 3, 5, and 10, respectively, then the appropriate training epochs were identified through an epoch-error plot. Each RNN model was trained three times and the most appropriate parameters of each RNN model were determined. We used testing set to compare the performance of each model and determine the optimal RNN model.

Firstly, the original data was normalized by the following formula, that is, all values were converted to the interval [0, 1],

$$X^{\prime }=\frac{X-X_{min}}{X_{max}-X_{min}},$$

where *X* are the values of original data, *X*_*max*_ is the maximum value of the original data, *X*_*min*_ is the minimum value of the original data, and *X*′ are the normalized values after conversion. Secondly, five different RNN models which did not incorporate air pollutants were constructed, by separately using the number of PTB cases in the previous month and the previous two, three, six, and twelve months as sequential inputs of the training set, and the number of PTB cases in the current month as the output of the training set. The performance of five RNN models were compared by using testing set and an optimal model was selected. Then, by Spearman rank correlation test, the correlations between PTB cases in the current month and air pollutants with a lag of 1 to 12 months were separately evaluated. Thirdly, those air pollutants (*P* < 0.05) which were significantly correlated with the PTB cases were incorporated into the optimal RNN model and the best lag order of the impact of each air pollutant on the PTB cases were determined. The optimal model incorporating air pollutants was finally determined, by using testing set to compare the performance of all RNN models.

### 2.4 Model assessment

#### 2.4.1 MAPE and RMSPE criteria

Prediction accuracy is an important criterion for evaluating forecasting validity. For such a reason, an error analysis based on two statistical measures, i.e. the Mean Absolute Percentage Error (MAPE) and Root Mean Square Percentage Error (RMSPE), is employed to estimate model performances and reliability [37]. The MAPE and the RMSPE are defined as

$$MAPE=\left(\frac{1}{n}\overset{n}{\underset{t=1}{\sum }}|\frac{x_{t}-{\hat{x}}_{t}}{x_{t}}|\right)\times 100\%,$$

$$RMSPE=\sqrt{\frac{\frac{1}{n}\sum_{t=1}^{n}{\left(\frac{x_{t}-{\hat{x}}_{t}}{x_{t}}\right)}^{2}}{n-1}}\times 100\%,$$

where *n* is the number of data, *x*_*t*_ and ${\hat{x}}_{t}$ are the actual and forecast values at time *t*, respectively. The criteria of MAPE and RMSPE are shown in Table 1.

**Table 1:** Criteria of MAPE and RMSPE.

MAPE and RMSPE	Forecasting Power
<10%	Highly accurate forecasting
10–20%	Good forecasting
20–50%	Reasonable forecasting
>50%	Inaccurate forecasting

#### 2.4.2 DISO index

Some single statistical indicators, such as Correlation coefficient (R), Absolute Error (AE), Root Mean Square Error (RMSE) and Mean Absolute Percentage Error (MAPE), were commonly used to evaluate the fitting accuracy of simulated models. Recently, DISO, a new comprehensive index was developed in [38, 39], which was used to evaluate overall model performance. It is a merge of different statistical metrics including R, AE, and RMSE according to the distance between the simulated model and observed field in a three-dimension space coordinate system. DISO is defined as follows:

$$DISO=\sqrt{{\left(1-R\right)}^{2}+NAE^{2}+NRMSE^{2},}$$

where R is Correlation coefficient, *NAE* and *NRMSE* are normalized *AE* and *RMSE*, respectively, its’ formulars are as follows:

$$R=\frac{\sum_{i=0}^{n}\left(a_{i}-\bar{a}\right)\left(b_{i}-\bar{b}\right)}{\sqrt{\sum_{i=0}^{n}{\left(a_{i}-\bar{a}\right)}^{2}}\sqrt{\sum_{i=0}^{n}{\left(b_{i}-\bar{b}\right)}^{2}}},$$

$$NAE=\frac{1}{\bar{a}n}\sum_{i=0}^{n}{\left(b_{i}-a_{i}\right)}^{2},$$

$$NRMSE=\frac{1}{\bar{a}}\sqrt{\frac{1}{n}\sum_{i=0}^{n}{\left(b_{i}-a_{i}\right)}^{2}},$$

here, assuming *a*_*i*_ (*i* = 1, 2, …, *n*) is the series of PTB cases and *b*_*i*_ (*i* = 1, 2, …, *n*) is the series of simulated model, $\bar{a}$ and $\bar{b}$ are the mean values of *a*_*i*_ and *b*_*i*_. The smaller values of DISO mean the higher accuracy by the model simulation.

## 3 Results

### 3.1 Descriptive statistics of PTB cases

**Fig 2:**
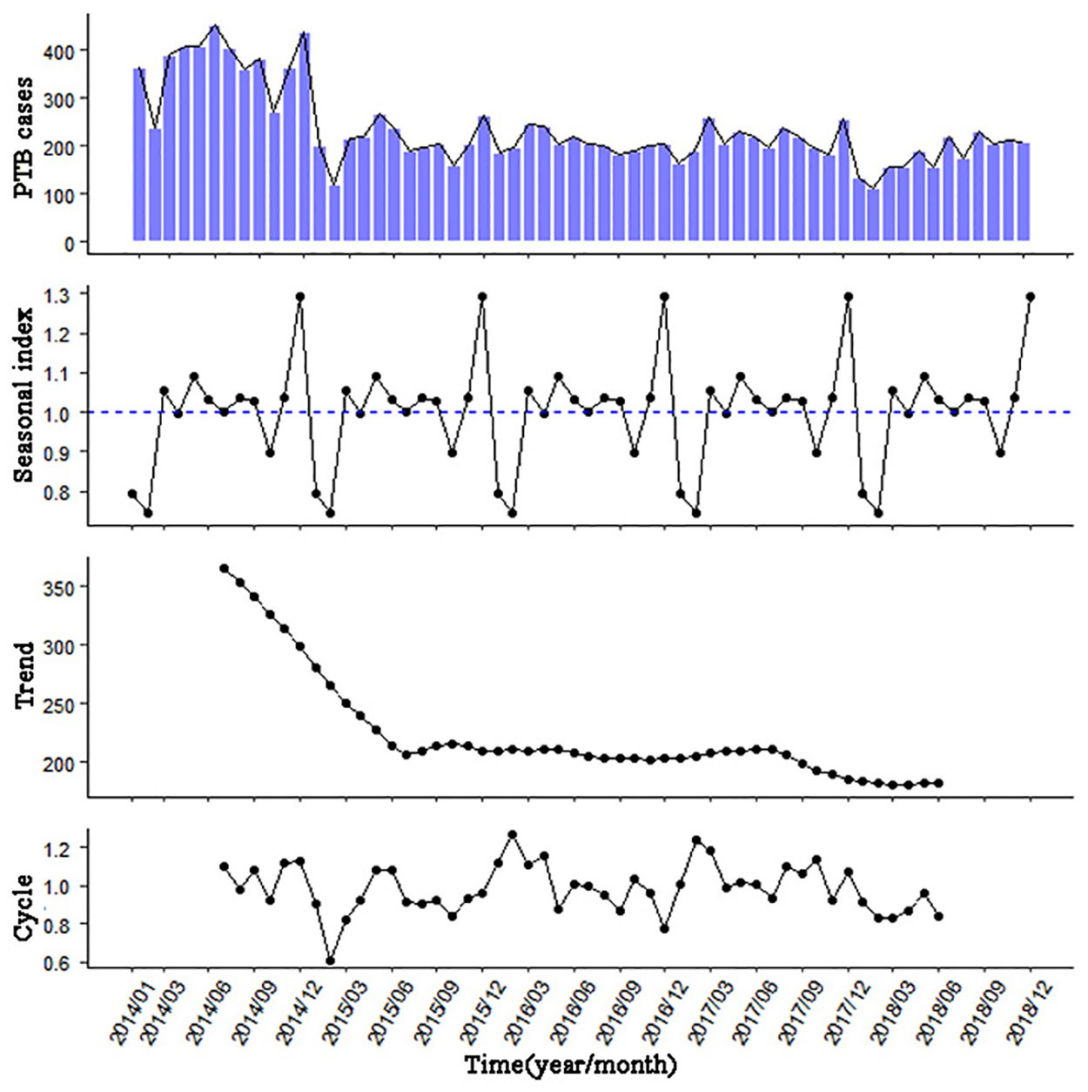
Decomposition of the number of PTB cases in Urumqi from January 2014 to December 2018.

During the study period from January 2014 to December 2018 (60 months), a total of 14151 PTB cases were included, with the average of 2830 cases per year and the maximum of 4470 cases in 2014.The total number of annual PTB cases in 2015 was considerably lower than that in 2014 by 44%, and then the changing trend was relatively gentle. In Fig 2, it was shown that the number of PTB cases in Urumqi had an obvious seasonal pattern and a long-term trend of gradual decrease. The seasonal index (or called season exponent), which can reflect a stable relationship between the average number of new monthly PTB cases and the average number of new total PTB cases, with a peak in annual October and a valley in February of the next year.

There were seasonal fluctuations and periodic trends of air pollutants in Urumqi, roughly showing the variation of single peak and single valley (S1 Fig). CO_2_, PM_2.5_, SO_2,_ and NO_2_ had similar seasonal patterns, with higher values occurring from December to January of the next year. The peak of the O_3_ occurred from May to June and the seasonal fluctuation of PM_10_ is unstable. In addition, the median of CO, PM_2.5_, PM_10_, NO_2_, SO_2,_ and O_3_ were 0.93 *μ*g/m^3^, 44 *μ*g/m^3^, 111 *μ*g/m^3^, 12.5 *μ*g/m^3^, 46 *μ*g/m^3^, and 63.5 *μ*g/m^3^, respectively (S1 Table).

### 3.2 Results of model discernment, parameters estimation, and model diagnosis

**Fig 3:**
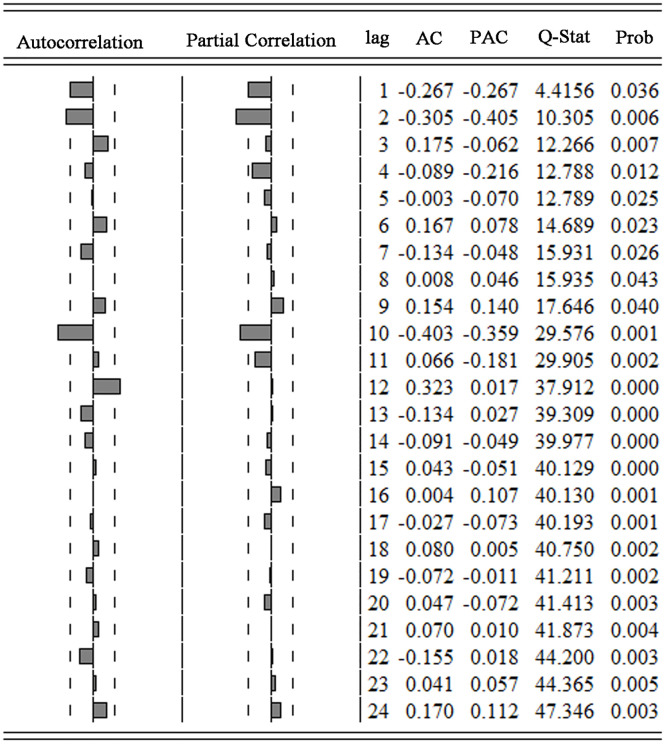
ACF and PACF diagrams after first-order difference of PTB cases in Urumqi.

As shown in Fig 2, it was obvious that the series of PTB cases in Urumqi was non-stationary. ACF diagram and PACF diagram were obtained after first-order difference (see Fig 3). The ACF diagram showed that the ACF values fall into double standard deviation intervals after lagging 2 orders. In conclusion, the series of PTB cases after first-order difference had a short-term correlation and it was stationary by the ADF test (*ADF* = −9.14, *P* < 0.05).

The model ARIMA (P, 1, q) (P, 0, q)_12_ was preliminarily determined by the data characteristics of the number of PTB cases and the process of stabilization. Next, in order to choose the optimal model in a larger range, the analysis of ACF and PACF was performed and showed *p*, *q*, *Q* = 0,1 or 2, *P* = 0 or 1 (see Fig 3), so there was a total of 3 × 3 × 3 × 2 = 54 different choices. T-tests for the coefficients of 54 models and Box tests for the residuals [24] were separately implemented. Finally, 10 models passed the test and their goodness-of-fit evaluation results were provided in Table 2 by using AIC, BIC, and MAPE criteria.

**Table 2:** The evaluations of goodness-of-fit for plausible ARIMA models.

Model	AIC	BIC	MAPE(%)
**ARIMA (0,1,1)×(0,0,1)**_**12**_	635.27	641.5	16.98
**ARIMA (1,1,0)×(0,0,1)**_**12**_	641.2	647.44	17.06
**ARIMA (1,1,2)×(0,0,1)**_**12**_	633.36	643.75	15.98
**ARIMA (2,1,0)×(0,0,1)**_**12**_	634.61	642.91	16.47
**ARIMA (1,1,2)×(0,0,0)**_**12**_	640.66	648.97	16.83
**ARIMA (2,1,0)×(0,0,0)**_**12**_	641.7	647.93	17.84
**ARIMA (0,1,1)×(1,0,0)**_**12**_	634.55	640.78	16.86
**ARIMA (1,1,0)×(1,0,0)**_**12**_	638.4	644.63	16.92
**ARIMA (1,1,2)×(1,0,0)**_**12**_	633.08	643.47	16.3
**ARIMA (2,1,0)×(1,0,0)**_**12**_	634.53	642.84	16.68

According to the criteria of minimum information, ARIMA (1,1,2)×(0,0,1)12 was the optimal model with the minimum values of BIC = 643.75, MAPE = 15.98% in 10 candidate models (see Table 2). The results of parameters estimation and white noise test of model ARIMA (1,1,2)×(0,0,1)_12_ were separately shown in Tables 3 and 4, and all *P*-values were statistically significant (*P* < 0.05).

**Table 3:** Parameters estimation for ARIMA (1,1,2)×(0,0,1)_12_ model.

Parameter	Coefficient	Standard error	T-value	*P*-value
**ar1**	-0.6977	0.1237	5.64	<0.001
**ma1**	0.3873	0.1266	3.06	0.0017
**ma2**	-0.6127	0.1162	5.27	<0.001
**sma1**	0.5972	0.1865	3.20	0.0011

**Table 4:** White noise test of residuals for ARIMA (1,1,2)×(0,0,1)_12_ model.

Model	lag	χ^2^	*P*-value
**ARIMA (1,1,2)×(0,0,1)**_**12**_	6	1.2741	0.9731
	12	8.9354	0.7084
	18	12.36	0.8281

As shown in Table 5, ARIMA models were developed for each air pollutant and the optimal models of each air pollutant were selected according to the AIC and BIC criteria, respectively.

**Table 5:** The optimal models for each air pollutant.

Air pollutants	Model	AIC	BIC
**PM**_**2.5**_	ARIMA (0,1,2)×(2,0,1)_12_	566.47	578.93
**PM**_**10**_	ARIMA (2,2,3)×(1,0,2)_12_	597.01	615.56
**NO**_**2**_	ARIMA (2,1,0)×(0,0,2)_12_	3.63	18.17
**SO**_**2**_	ARIMA (0,2,1)×(1,0,1)_12_	449.07	457.31
**CO**	ARIMA (1,1,1)×(0,0,3)_12_	48.21	60.68
**O**_**3**_	ARIMA (1,2,2)×(0,1,1)_12_	343.81	352.96

### 3.3 The results of air pollutants inclusion

**Fig 4:**
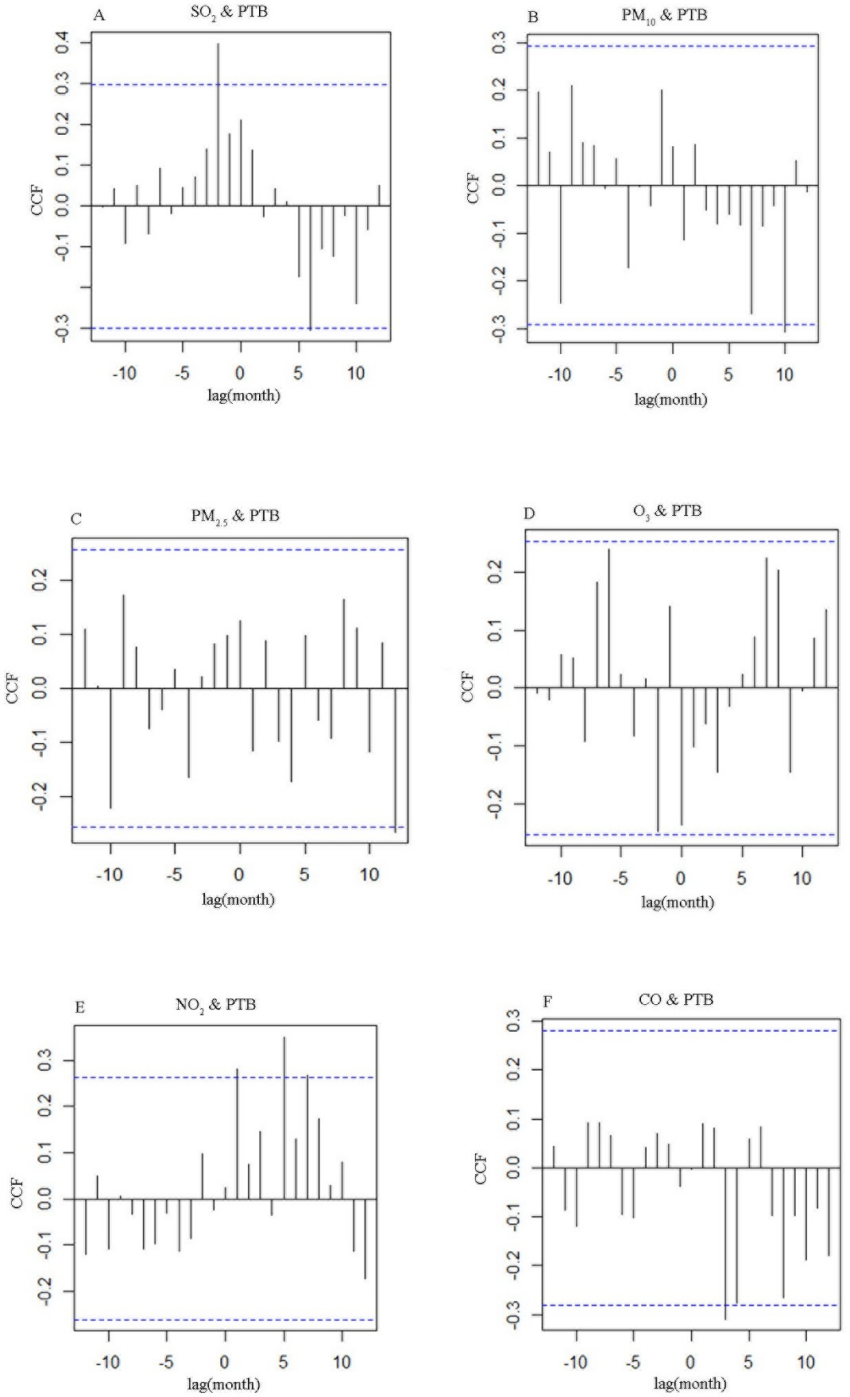
Cross-correlations between the pre-whitened PTB cases and air pollutants.

In order to investigate the correlation between PTB cases and each air pollutant at different lag times, we will find the best multivariate model. Hence, we considered all air pollutants (PM_2.5_, PM_10_, SO_2_, CO, NO_2,_ and O_3_) as regression variables in the ARIMA (1,1,2)×(0,0,1)_12_ model. As shown in Fig 4, there were significant correlations between PM_2.5_, PM_10_, NO_2_, SO_2_, CO, and the PTB cases, except for O_3_ (see Fig 4D). More specifically, the monthly average of SO_2_ at a lag of 6 months, the monthly average of PM_10_ at a lag of 10 months and the monthly average of PM_2.5_ at a lag of 12 months, the monthly average of NO_2_ at a lag of 1 month or 5 months, the monthly average of CO at a lag of 3 months were significantly related to the number of PTB cases.

In the following, these five relative air pollutants SO_2_, PM_10_, PM_2.5_, NO_2,_ and CO were included in the multivariate ARIMA model to establish the corresponding ARIMAX models. Only three of the seven ARIMAX models passed the residual and parameter tests, and their AIC and MAPE values were calculated, respectively (see Table 6). As shown in Table 6, the values of AIC and MAPE of ARIMAX models included air pollutants were lower than the ARIMA model. In particular, ARIMAX (1,1,2)×(0,1,1)_12_+PM_2.5_ with 12-month lag has the smallest AIC value (AIC = 479.32) and MAPE value (MAPE = 6.766%), which was the optimal ARIMAX model.

**Table 6:** The residual and parameter tests of ARIMAX models.

Model	lag	Coefficient		Ljung-Box test		AIC	MAPE (%)
		T	*P*-value	χ^2^	*P-*value		
**ARIMA(1,1,2)**×**(0,1,1)**_**12**_	—	—	—	1.27	0.97	633.36	15.989
**ARIMA(1,1,2)**×**(0,1,1)**_**12**_**+NO**_**2**_	7	5.42	<0.001	3.19	0.78	514.66	13.319
**ARIMA(1,1,2)**×**(0,1,1)**_**12**_**+SO**_**2**_	6	2.49	0.001	4.53	0.6	506.47	12.728
**ARIMA(1,1,2)**×**(0,1,1)**_**12**_**+PM**_**2.5**_	12	3.46	0.001	5.8	0.45	479.32	6.766

### 3.4 RNN model

**Fig 5:**
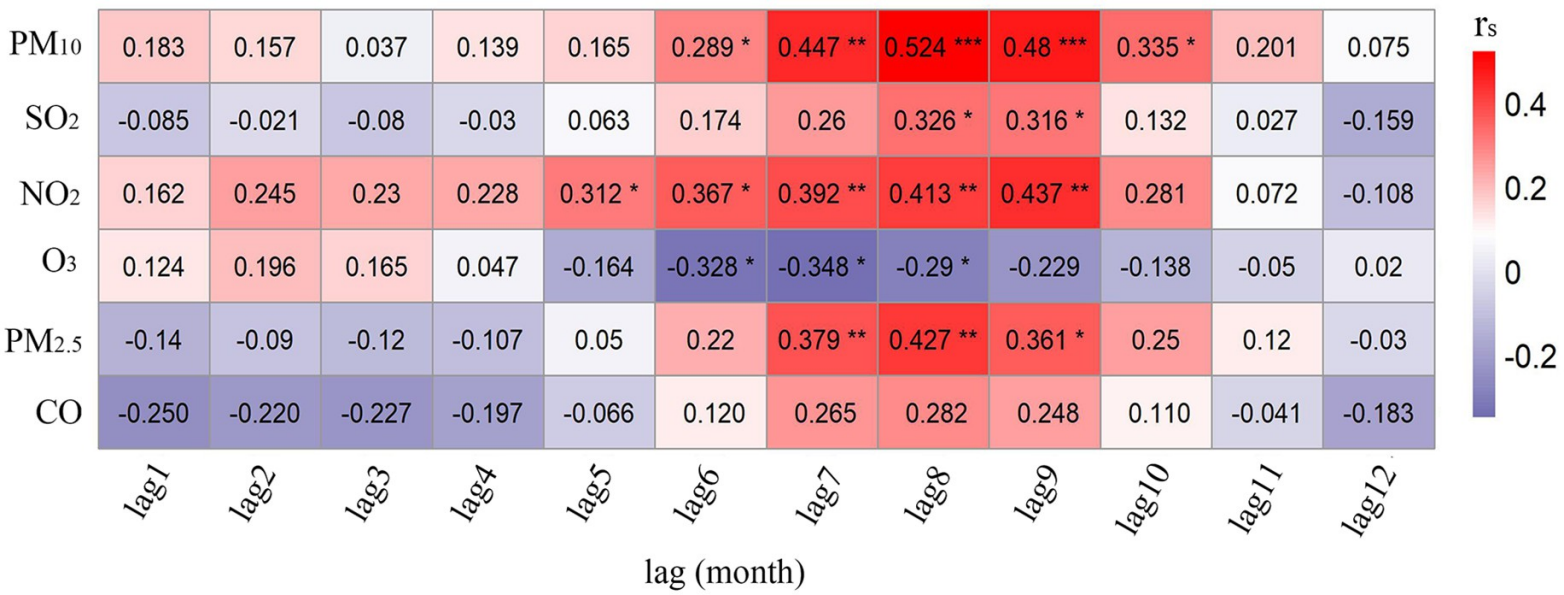
Spearman ranks correlation coefficients between the PTB cases and air pollutants with a lag of 1 to 12 months. Notes *: *P* < 0.05 **: *P* < 0.01 ***: *P* < 0.001.

**Fig 6:**
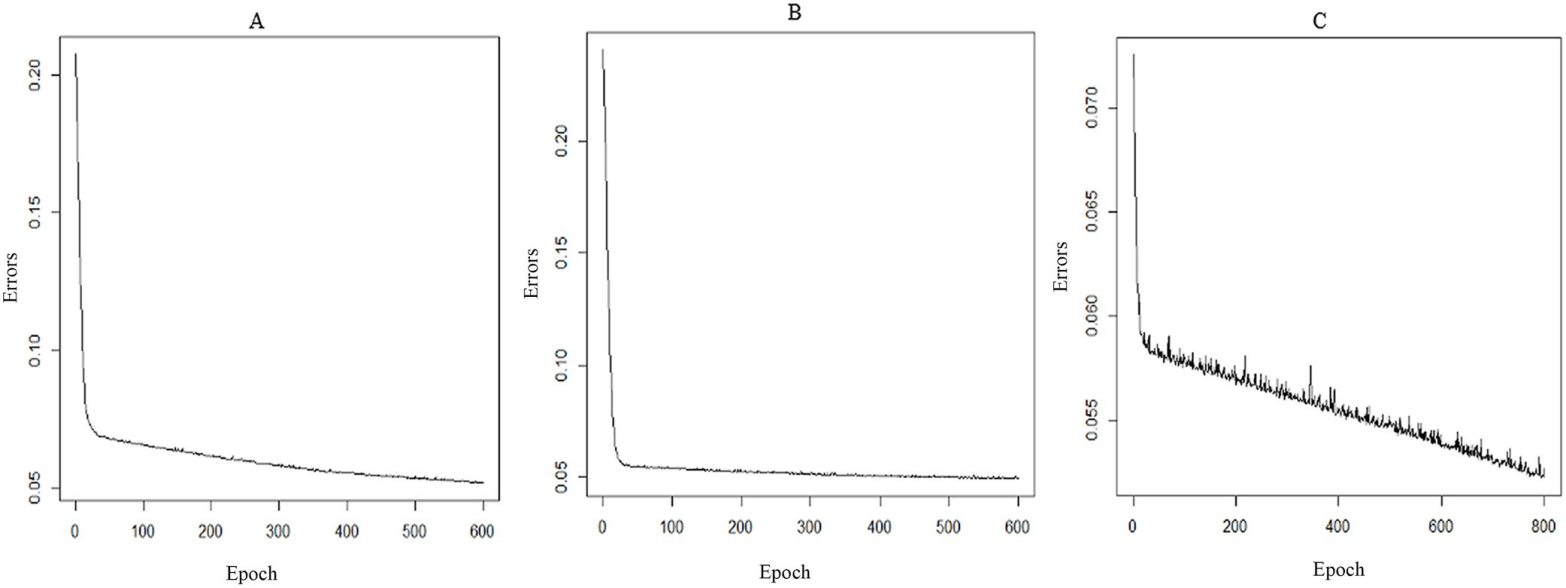
Epoch-error plots of the RNN9 after three training cycles. (A) First cycle, (B) Second cycle, (C) Third cycle.

Firstly, the appropriate parameters of each RNN model were identified by comparing the MAPE values. It was found that RNN5 model had the smallest MAPE value (see Table 7), which implied RNN5 model was the optimal one. Apart from CO, other air pollutants in different lag orders (O_3_, PM_2.5_, PM_10_, SO_2_, and NO_2_) had significant correlations with the PTB cases (see Fig 5). Then, air pollutants O_3_, PM_2.5_, PM_10_, SO_2_, and NO_2_ were incorporated into RNN5 model to construct other RNN models (RNN6~RNN10). As shown in Table 8, the smallest MAPE value in RNN6-RNN10 models separately were RNN6(RNN5+PM_10_(lag8)), RNN7(RNN5+SO_2_(lag8)), RNN8(RNN5+O_3_(lag7)), RNN9(RNN5+PM_2.5_(lag8)), RNN10(RNN5+NO_2_(lag8)). Thirdly, comparing results of the 10 models in Tables 7 and 8 found that RNN9 (RNN5+PM_2.5_(lag8)) model was determined the optimal RNN model with the smallest MAPE (6.29%). As shown in Fig 6, the downward trend in epoch-error plots of RNN9 after three training cycles was no longer significant after reaching the set number of epochs, which indicated that the training epochs of RNN9 were appropriate.

**Table 7:** Training results of RNN1-RNN5 models.

Model	Learning rate	Dimensions of hidden layer	Number of epochs	MAPE^1^ (%)	MAPE^2^ (%)	MAPE^3^ (%)
**RNN1**	0.2	10	60	17.71	18.44	17.26
**RNN2**	0.05	10	250	17.45	16.37	16.57
**RNN3**	0.1	10	150	21.51	20.66	21.03
**RNN4**	0.1	10	150	10.50	10.93	10.78
**RNN5**	0.1	3	400	10.47	10.51	10.76

Notes MAPE^1^, MAPE^2^, and MAPE^3^ represent the MAPE values after first, second and third training of the model, respectively.

**Table 8:** Training results of RNN models with incorporating air pollutants (O_3_, PM_2.5_, PM_10_, SO_2_, and NO_2_).

Model		Learning rate	Dimensions of hidden layer	Number of epochs	MAPE^1^ (%)	MAPE^2^ (%)	MAPE^3^ (%)
RNN6	RNN5+PM_10_(lag6)	0.2	10	150	7.36	8.36	9.15
	RNN5+PM_10_(lag7)	0.2	10	150	8.75	8.92	8.06
	RNN5+PM_10_(lag8)	0.2	3	250	6.94	7.08	7.17
	RNN5+PM_10_(lag9)	0.1	10	250	7.73	7.94	8.14
	RNN5+PM_10_(lag10)	0.05	3	1000	8.33	8.03	8.04
RNN7	RNN5+SO_2_(lag8)	0.05	10	600	6.81	6.30	8.20
	RNN5+SO_2_(lag9)	0.05	10	600	7.37	7.91	8.74
RNN8	RNN5+O_3_(lag6)	0.1	10	250	8.74	7.77	8.11
	RNN5+O_3_(lag7)	0.05	10	600	7.17	7.32	6.26
	RNN5+O_3_(lag8)	0.05	3	1000	8.81	7.78	8.59
RNN9	RNN5+PM_2.5_(lag7)	0.05	3	800	8.64	8.04	8.65
	RNN5+PM_2.5_(lag8)	0.05	3	800	6.74	6.42	6.29
	RNN5+PM_2.5_(lag9)	0.2	5	200	8.05	8.33	7.95
RNN10	RNN5+NO_2_(lag5)	0.05	5	800	9.23	9.22	8.45
	RNN5+NO_2_(lag6)	0.1	5	400	8.57	8.20	7.83
	RNN5+NO_2_(lag7)	0.2	3	250	8.05	7.92	8.27
	RNN5+NO_2_(lag8)	0.2	5	200	7.59	6.89	7.78

Notes MAPE^1^, MAPE^2^, and MAPE^3^ represent the same meanings in Table 6.

Fig 6 shows the plots of the training errors function of the PTB cases prediction model changing with the number of iterations, and it can be seen that RNN model tends to be stable (with the error value < 0.05) when the number of trainings reaching 600, which indicates that the prediction performance is better.

### 3.5 Results of ARIMA, ARIMAX, and RNN model assessment

**Fig 7:**
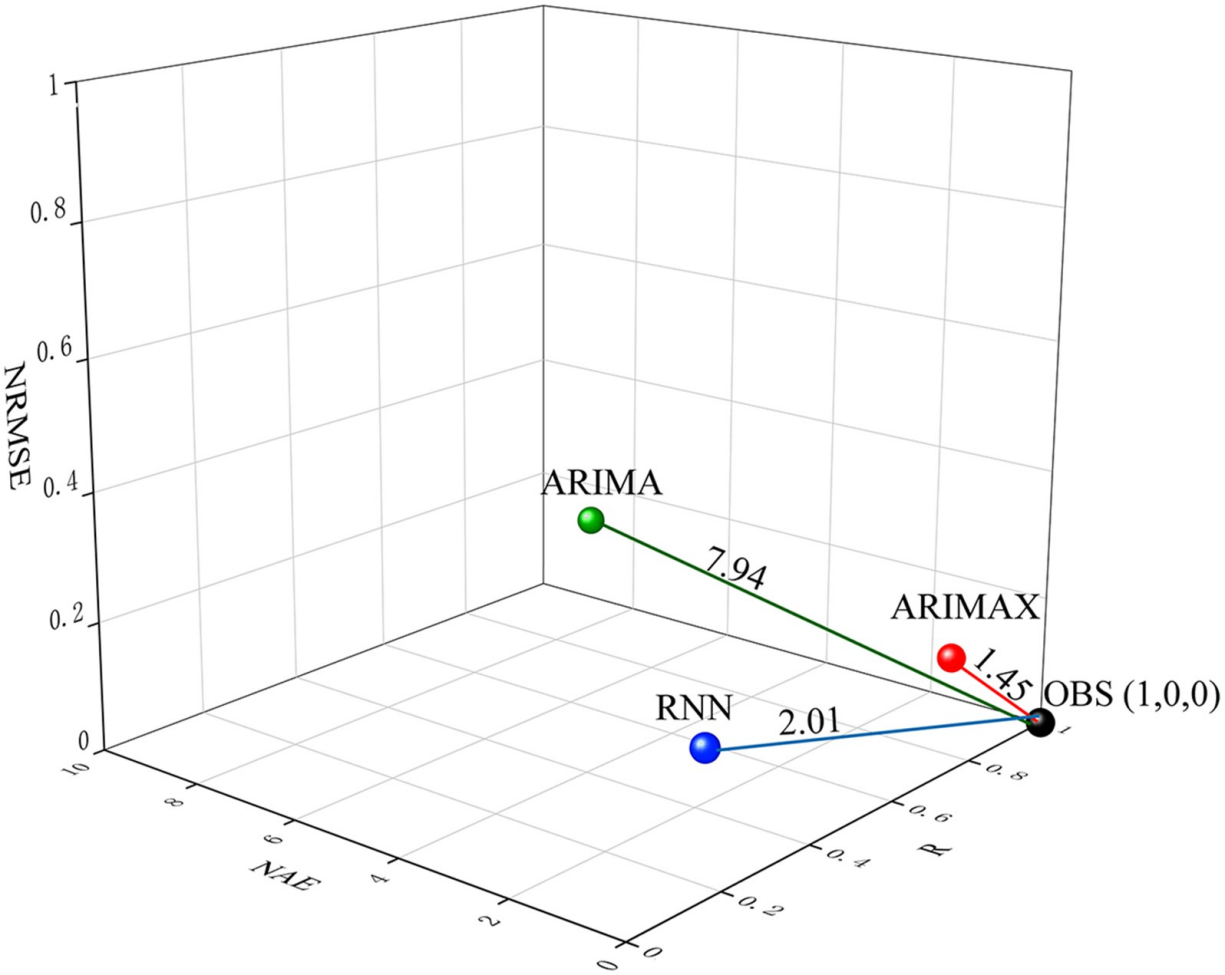
Distances between OBS and simulation results by ARIMA, ARIMAX, and RNN models.

As shown in Fig 7, the comprehensive accuracies of the ARIMA, ARIMAX and RNN models are quantitatively measured by the DISO with the values of 7.94, 1.45, and 2.01, respectively. It was implied that ARIMAX model was the optimal one superior to the ARIMA and RNN models. Therefore, in the following, ARIMAX model was applied to predict the PTB cases in Urumqi from January to December 2019.

### 3.6 Fitting and predicting results of models

**Fig 8:**
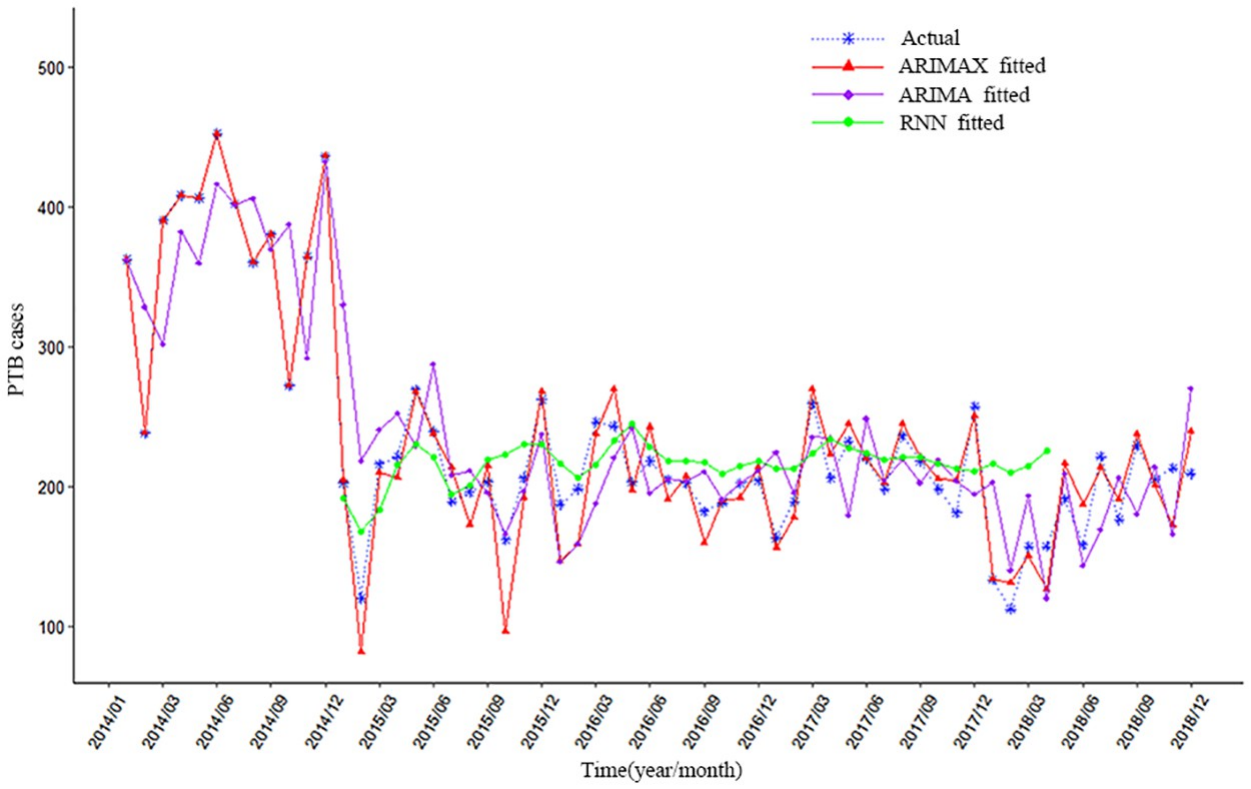
The fitting results of ARIMA, ARIMAX, and RNN models.

The optimal models ARIMA (1,1,2)×(0,0,1)_12_, ARIMAX (1,1,2)× (0,1,1)_12_+PM_2.5_(lag12) and RNN9(RNN5+ PM_2.5_(lag8)) were applied to fit PTB cases from January 2014 to December 2018. As can be seen from Fig 7, it was found that ARIMAX model is good in data fitting (see Fig 8), superior to ARIMA and RNN models (especially from January 2014 to June 2015), which shown that ARIMAX model had the best prediction performance.

**Fig 9:**
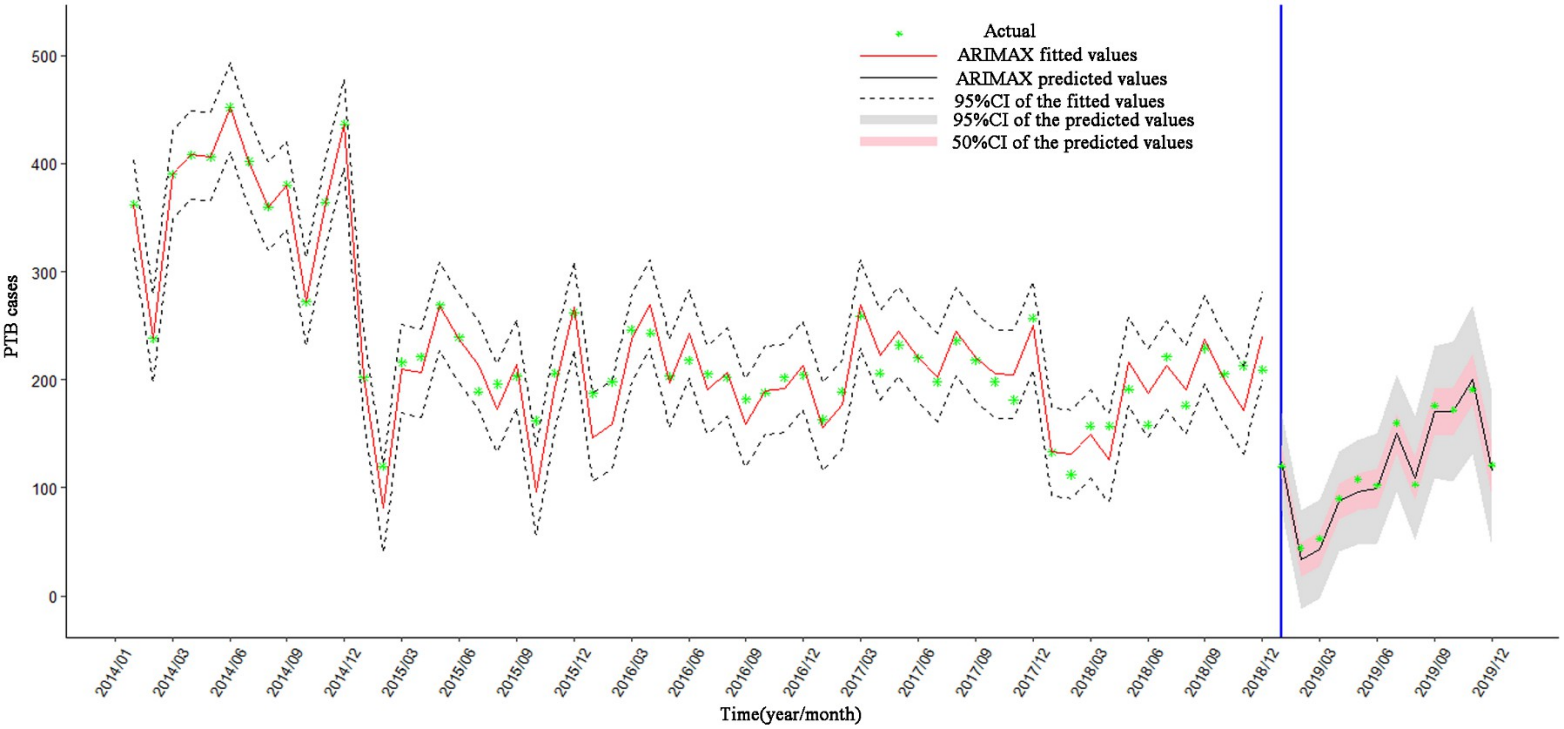
The fitting and predicting results of the ARIMAX(1,1,2)×(0,1,1)_12_+PM_2.5_(lag = 12)model.

Hence, ARIMAX (1,1,2) × (0,1,1)_12_ + PM_2.5_ (lag = 12) model was employed for predicting PTB cases from January 2019 to December 2019. As shown in Fig 9, the predicted values of the model were in good agreement with the actual values of the number of PTB cases. and showed a decrease obviously in 2019, with a trend of cycle fluctuations consistent with previous years. The results of evaluating forecasting validity of ARIMAX (1,1,2)×(0,1,1)_12_+PM_2.5_ (lag = 12) model was shown that MAPE = 0.75%, RMSPE = 10.72% (According to Table 1), it was indicated that the ARIMAX(1,1,2)×(0,1,1)_12_+PM_2.5_ (lag = 12) model has high accurate forecasting power.

## 4 Discussions

It is well known that air pollution is a global health threat. Although the bronchopulmonary tract has multiple protective mechanisms, air pollution can still harm acutely for respiratory system. Relevant results [40, 41] have shown that the concentration of air pollutants has been linked with clinical manifestations of pulmonary diseases and it is associated with morbidity and mortality induced by respiratory diseases.

In this paper, the impact of air pollutants (CO, PM_2.5_, PM_10_, SO_2_, O_3,_ and NO_2_) on the number of PTB cases in Urumqi was investigated by using ARIMA, ARIMAX, and RNN models. The results of the cross-correlation analysis showed that apart from O_3_, other air pollutants (PM_2.5_, PM_10_, SO_2_, CO, and NO_2_) all had a lagged effect on the PTB cases in Urumqi, which is consistent with the findings in [42]. Specifically, PM_2.5_ had a lag (12 months) impact on the number of PTB cases in Urumqi. This may be due to the fact that PM_2.5_ can enter the fine bronchi and alveoli of the lung through the respiratory tract, and increased secretion and susceptibility of the respiratory mucosa thereby leading to the obstruction of the mucus-cilia clearance mechanism. Another potential explanation might be that, when a large amount of PM_2.5_ is inhaled into the lung through the respiratory tract, macrophages will produce a huge number of bioactive factors acting on PM_2.5_ and release inflammatory factors to damage the tissue structure of the lung, which may result in inflammatory lesions in the lungs. Both processes are slow, which could lead to a lagged effect of PM_2.5_ on the development of PTB. The result in [43] also showed that PM_2.5_ has a certain chronic health risk for humans in Urumqi.

It can also be seen from the results of this paper that those three models (ARIMA, ARIMAX and RNN model) have different merits in data analysis. For example, ARIMA model is adept at identifying hidden trends (such as autocorrelation, and seasonal variation) in a dataset. ARIMA could capture behaviors of both stationary and non-stationary series and describe the linear relationship between disease incidence and predictors, but its predictive ability is limited by reliance on prior knowledge of parameters or inherent time-lag and it is not account for additional factors which influence the occurrence and development of PTB. Different from ARIMA model, ARIMAX model could deal with multivariate time series data by adding other variables related to the PTB cases series to improve the prediction accuracy. However, the essence of ARIMA and ARIMAX is linear and it is insufficient to fit the complex multivariable dependencies, RNN model with a strong nonlinear fitting ability can overcome this limitation. Moreover, RNN retains more long-term sequence information and has memory to store the values that have been calculated.

This paper also has several limitations. Firstly, ARIMAX model is dependent on a large amount of historical data and requires the data to remain relatively stable, so as to achieve accurate and effective prediction. If the external factors suddenly change or new variables are introduced, the prediction effect of the model will be affected and thus the prediction performance will be reduced. In order to achieve more accurate prediction, ARIMAX model can be combined with differential equation models, regression analysis models, gray prediction models, artificial neural networks and other models to propose a combination model of time series analysis. Furthermore, the corresponding combined models can be built to obtain more accurate prediction by considering meteorological factors, economic factors, and other factors that have an impact on PTB.

## 5 Conclusion

In this paper, by using the ARIMA model, a multivariate time series ARIMAX and RNN model, the impact of air pollutants (O_3_, PM_2.5_, PM_10_, SO_2_, CO, and NO_2_) on the number of PTB cases in Urumqi was investigated. It was found that ARIMAX model is obviously good in data fitting, superiorly to ARIMA and RNN models (especially from January 2014 to June 2015), which has also been confirmed by the result that ARIMAX model had the smallest DISO value by comparing with those of the other two models. Therefore, ARIMAX (1,1,2)×(0,1,1)_12_+PM_2.5_ with 12-month lag was applied to predict the number of PTB cases from January to December 2019 in Urumqi. The predicted results of the ARIMAX model were in good agreement with the actual PTB cases, which presented that ARIMAX model had high accurate forecasting power and was applicable for predicting PTB cases in Urumqi. Moreover, the predicting results suggested that PTB cases declined obviously. It may be related to the comprehensive coverage of DOTS strategy and the implementation of universal health checkups in Urumqi, which make more PTB patients without discharge of bacterium to be earlier detected and diagnosed. Additionally, the centralized hospitalization of PTB patients in the infectious stage and the plan of "centralized medication + nutritional breakfast" for PTB patients have been carried out in Urumqi, which would effectively promote the recovery of PTB patients and reduce the spread of tuberculosis. A series of the adjustment of energy structure has improved air quality in Urumqi, such as “coal to gas conversion” and the "Blue Sky Project", which would reduce the emission of PM_2.5_, PM_10_, and other air pollutants thus decreasing the risk of PTB.

## Supporting information

S1 Table Description of the monthly air pollutants from 2014 to 2018. (DOCX)

S1 Fig Time series plots of the six air pollutants in Urumqi. (TIF)

S1 File R code of ARIMA model. (R)

S2 File R code of ARIMAX model. (R)

S3 File R code of RNN model. (R)

S1 Data (XLSX)
