# Supplementary material for: A comparative study of three models to analyze the impact of air pollutants on the number of pulmonary tuberculosis cases in Urumqi, Xinjiang
Source: PLoS One. 2023 Jan 17;18(1):e0277314. doi: 10.1371/journal.pone.0277314 (PMC9844834; doi:10.1371/journal.pone.0277314)
Supplement: S1 Table — (DOCX) [file pone.0277314.s001.docx]

Table Supplementary S1. Description of the monthly air pollutants from 2014 to 2018

| Variables | Mean | SD | Min | P_25_ | P_50_ | P_75_ | Max |
| --- | --- | --- | --- | --- | --- | --- | --- |
| CO | 1.37 | 0.84 | 0.53 | 0.72 | 0.93 | 1.94 | 3.45 |
| PM_2.5_ | 63.9 | 51.33 | 18 | 25 | 44 | 90.5 | 226 |
| PM_10_ | 118.72 | 54.85 | 50 | 75.2 | 111 | 147.5 | 287 |
| NO_2_ | 16.12 | 10.69 | 6 | 9 | 12.5 | 17.5 | 50 |
| SO_2_ | 49.95 | 16.37 | 28 | 37 | 46 | 60 | 90 |
| O_3_ | 64.87 | 33.68 | 14 | 35 | 63.5 | 94.75 | 124 |
