# Supplementary figures and images for: A comparative study of three models to analyze the impact of air pollutants on the number of pulmonary tuberculosis cases in Urumqi, Xinjiang
Source: PLoS One. 2023 Jan 17;18(1):e0277314. doi: 10.1371/journal.pone.0277314 (PMC9844834; doi:10.1371/journal.pone.0277314)

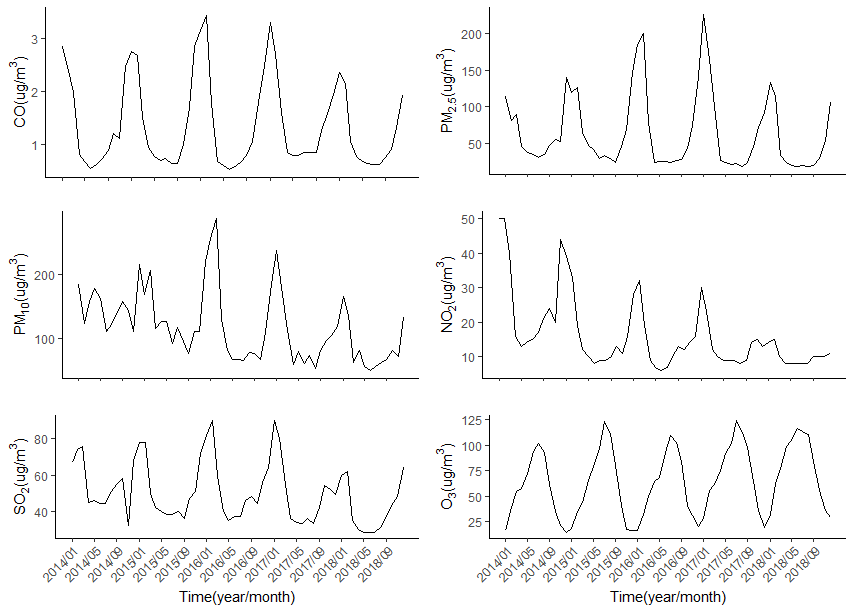

Supplement: S1 Fig — (TIF) [file pone.0277314.s002.tif]
